# Synergistic immune interactions between T cells and natural killer cells in allogeneic haematopoietic stem cell transplantation for acute myeloid leukaemia: current status and future directions

**DOI:** 10.1080/07853890.2025.2601404

**Published:** 2026-02-02

**Authors:** Nan Wang, Hanxue Zheng, Zhengdong Hao, Pingling Yin, Liansheng Zhang, Lijuan Li

**Affiliations:** ^a^ The Second Hospital & Clinical Medical School, Lanzhou University, Lanzhou, China; ^b^ Gansu Provincial Clinical Medical Research Center for Hematological Diseases, Lanzhou, China; ^c^ Pingliang City People’s Hospital, Pingliang, China

**Keywords:** Acute myeloid leukaemia, allogeneic haematopoietic cell transplantation, graft-versus-leukaemia effect, graft-versus-host disease, T cells, NK cells

## Abstract

**Background:**

Acute myeloid leukaemia (AML) is a highly heterogeneous haematologic malignancy. Current therapeutic strategies include chemotherapy, targeted therapy and haematopoietic cell transplantation (allo-HSCT) and autologous haematopoietic cell transplantation (auto-HSCT). The graft-versus-leukaemia (GVL) effect and graft-versus-host disease (GVHD) in allo-HSCT remain major research foci, with emerging evidence highlighting the synergistic roles of T cells and natural killer (NK) cells in allo-HSCT immunity. This review systematically integrates the cooperative immunological interactions between T cells and NK cells and elucidates their critical significance in post-transplant immunotherapy.

**Methods:**

This review systematically summarizes the cytotoxic mechanisms, immune reconstitution processes and related immunotherapeutic approaches involving T cells and NK cells in AML in the context of allo-HSCT and further elucidates their unique role in post-transplant immune regulation from the perspective of coordinated T-cell and NK-cell interactions.

**Results:**

T cells and NK cells exert synergistic effects in post-transplant immune reconstitution, GVL responses, GVHD regulation and subsequent immunotherapeutic interventions. Early NK-cell reconstitution provides a critical window for the restoration of T-cell function, whereas cytokines derived from T cells, such as IL-2 and IL-15, further enhance NK-cell activity. This dynamic immunological interplay not only shapes the balance between GVL and GVHD, but also informs the development of post-transplant immunotherapeutic strategies.

**Conclusion:**

The dynamic interplay between T cells and NK cells plays a pivotal role in allo-HSCT for AML. This review systematically integrates the cooperative functions of T cells and NK cells within the allo-HSCT immune landscape, offering new perspectives for advancing post-transplant immunotherapy. A deeper understanding of these mechanisms is expected to provide a theoretical foundation for optimizing post-transplant immune interventions in AML patients and for developing more precise therapeutic strategies.

## 1. Introduction

Acute myeloid leukaemia (AML) is an aggressive haematologic malignancy, and allogeneic haematopoietic stem cell transplantation (allo-HSCT) remains one of the most effective curative strategies currently available [1–3]. Allo-HSCT eliminates leukaemic cells through myeloablative chemotherapy or radiotherapy, while its long-term efficacy relies on donor-derived graft-versus-leukaemia (GVL) effect, which eradicates minimal residual disease (MRD) and reduces relapse risk [4,5]. However, donor immune cells also attack normal tissues, leading to graft-versus-host disease (GVHD). GVL and GVHD frequently coexist, forming the central paradox of allo-HSCT: immunosuppression aimed at controlling GVHD may simultaneously compromise GVL, posing a major clinical challenge [5–8]. Nevertheless, the GVL effect remains the cornerstone of allo-HSCT success and critically influences long-term prognosis in patients with AML. Among the principal mediators of GVL, T cells and natural killer (NK) cells have received substantial attention. Their coordinated roles in post-transplant immune reconstitution, the GVL response and the mitigation of GVHD offer new perspectives for the development of post-transplant immunotherapeutic strategies.

With advances in transplantation techniques and supportive care, transplant-related mortality has decreased, however, disease relapse has emerged as the predominant cause of post-transplant treatment failure [4,9]. Statistics indicate that relapse occurs in up to 30% of AML patients undergoing allo-HSCT, and the prognosis after relapse is extremely poor [10]. Therefore, maximizing donor immune-mediated GVL to prevent relapse while controlling GVHD has become a central focus of current research. This review summarize the cytotoxic mechanisms, immune reconstitution dynamics, contemporary immunotherapeutic strategies and synergistic interactions of T cells and NK cells in AML allo-HSCT, while providing perspectives for future research directions.

## 2. T cells in AML allogeneic haematopoietic stem cell transplantation

### 2.1. Mechanisms of the GVL effect

#### 2.1.1. The central role of T cells in the GVL effect

Donor-derived T cells are the central mediators of the GVL effect following transplantation in AML. During transplantation, infused donor T cells recognize and attack residual leukaemic cells in the recipient, a process that primarily depends on disparities in histocompatibility antigens between donor and recipient. Established antigenic targets include minor histocompatibility antigens (miHAs; e.g. HA-1, HA-2) and leukaemia-associated antigens such as WT1 and PRAME [11–13]. Alloreactive T cells recognize non-self antigens encoded by patient-specific genomic polymorphisms. In human leukocyte antigen (HLA)-matched allo-HSCT, GVL and graft-versus-tumour (GVT) responses are predominantly induced by donor T cells recognizing miHAs—polymorphic peptides encoded by SNPs within self-HLA molecules—arising from genetic differences between donor and recipient [14–16]. These antigens are processed by recipient- or donor-derived antigen-presenting cells and presented via major histocompatibility complex (MHC) class I or II molecules to donor T cells, triggering immune recognition and cytotoxic responses [13,17].

Donor T cells recognize heterogeneous antigenic peptides presented on leukaemic cells through the T-cell receptor (TCR). Antigen engagement triggers rapid T-cell activation and clonal expansion, followed by the release of cytotoxic effector molecules such as perforin and granzymes. Perforin disrupts membrane integrity by forming pores, enabling granzymes to enter the cytoplasm, where they induce DNA damage, mitochondrial dysfunction and the initiation of apoptotic cascades [18]. Additionally, activated T cells express Fas ligand (FasL), which engages Fas receptors on leukaemic cells to activate downstream caspase cascades, further promoting apoptosis [19,20]. T cells may also upregulate tumour necrosis factor (TNF) family members such as TRAIL, activating alternative apoptotic pathways to complement perforin- granzyme-mediated cytotoxicity [21].

Beyond direct cytotoxicity, donor T cells indirectly augment antitumour immunity through the release of pro-inflammatory cytokines, including interferon-γ (IFN-γ) and TNF-α. IFN-γ upregulates MHC class I expression on AML cells, enhancing antigen presentation, and simultaneously activates local antigen-presenting cells such as dendritic cells, thereby amplifying the antileukaemic immune response [22,23].

#### 2.1.2. The roles of different T-cell subsets in GVL

Distinct T-cell subsets also play differential roles in GVL. CD8^+^ T cells represent key cytotoxic effectors against leukaemic cells [24]. A study analysing bone marrow T lymphocytes from six post-allo-HSCT AML patients by single-cell RNA sequencing (scRNA-seq) revealed that patients achieving long-term remission had enriched CD8^+^ T cells and γδ T cells, with CD8^+^ T cells exhibiting high expression of cytotoxicity-associated genes [24], underscoring their importance in GVL. CD4^+^ T cells, on the other hand, provide critical helper functions, producing cytokines such as IL-2 and IL-21 that promote CD8^+^ T-cell activation and expansion, thereby strengthening the overall antileukaemic immune response [25,26]. Certain CD4^+^ subsets, such as Th1 cells, also demonstrate direct cytotoxicity via TNF-α and IFN-γ secretion, inducing apoptosis and clearance of leukaemic cells [26]. Moreover, memory T cells contribute to long-term tumour immunosurveillance due to their rapid expansion capacity and sustained survival [27,28]. In a phase II clinical trial, infusion of memory T cells after transplantation enhanced immune protection without increasing GVHD risk [29]. Another study tracking persistent T-cell clones post-allo-HSCT found that clonal populations were almost exclusively composed of CD8^+^ effector memory T cells (CD8TEM) [30], further supporting their critical role in GVL and immune surveillance.

#### 2.1.3. The roles of donor T cells in GVL and GVHD

Clinical observations reinforce the pivotal role of donor T cells in GVL. Guo et al. [31] demonstrated in both murine models and clinical cohorts that higher T-lymphocyte levels correlated with stronger GVL effects. However, other studies indicated that elevated donor T-cell numbers were associated with increased incidence of acute GVHD [32]. Conversely, clinical trials have shown that lower T-cell doses in grafts reduced GVHD but markedly increased relapse risk and worsened long-term survival [33–35]. These paradoxical findings highlight the immunological interconnection and delicate balance between GVL and GVHD. In MHC-mismatched transplantation, T cells respond to allogeneic MHC molecules, whereas in MHC-matched transplantation, they react to miHAs. Following activation and proliferation, these T cells differentiate into CD4^+^ and CD8^+^ subsets, both of which contribute to tissue damage during the effector phase of GVHD through the FasL/Fas apoptotic pathway or the perforin–granzyme cytolytic mechanism. Costimulatory signalling through the interaction of B7 and CD28 further enhances T-cell activation [36]. In T cells, miR-146a targets TRAF6 and regulates NF-κB-dependent transcription of TNF-α, thereby mitigating GVHD [37]. CD4^+^ T cells can further differentiate into Th1, Th2 and Th17 subsets. Th1 cells migrate to the gastrointestinal tract and liver via CCR9 and CCR5, where their production of IFN-γ contributes to tissue injury. Th2 cells migrate to the lungs through CCR4 and secrete IL-4, IL-5 and IL-13, exacerbating pulmonary damage and leading to fibrosis. Th17 cells secrete IL-17 and play a key role in cutaneous GVHD [38,39].

Recently, Liu et al. [40] identified a novel T-cell subset—PD-1^+^CD8^+^ stem cell memory T (TSCM)-like regulatory cells. Single-cell transcriptomic analysis demonstrated that these cells exhibit transcriptional features of stem cell memory T cells and possess both Treg and Teff activities. Notably, greater numbers of donor peripheral PD-1^+^CD8^+^ TSCM-like regulatory cells induced by G-CSF correlated with reduced GVHD incidence. These cells expressed higher levels of TGF-β under G-CSF stimulation, and both before and after mobilization exhibited cytotoxic proteins such as CD107a, perforin and granzyme B, indicating dual immunoregulatory and cytotoxic functions. *In vivo* studies further confirmed that grafts enriched with PD-1^+^CD8^+^ TSCM-like regulatory cells promoted superior GVL effects while limiting acute GVHD, thereby facilitating immune reconstitution and improving post-transplant survival. This discovery provides new avenues to maximize GVL while minimizing GVHD risk, offering hope for extended survival in transplant recipients.

In summary, the GVL effect in AML transplantation arises from multiple mechanisms synergistically mediated by donor T cells, including direct cytotoxicity, cytokine modulation and cooperation among diverse T-cell subsets. Nevertheless, the substantial mechanistic overlap between GVL and GVHD continues to pose a major challenge. Achieving selective enhancement of antigen-specific GVL while minimizing GVHD remains a central objective that will shape ongoing and future research efforts in the field.

### 2.2. Immune reconstitution and influencing factors

#### 2.2.1. T-cell immune reconstitution after allo-HSCT

Following allo-HSCT, the reconstitution of a functional donor-derived immune system is essential for sustaining the GVL effect and preventing infections. As the central component of adaptive immunity, T cells undergo a distinct, phased process of immune reconstitution. In the early stage, recovery mainly relies on the peripheral expansion of mature T cells infused with the graft [41,42], followed months later by the generation of new naive T cells derived from donor haematopoietic stem cells developing in the thymus, referred to as thymus-dependent reconstitution [43–45]. CD8^+^ T cells and effector memory T cells can partially reconstitute within weeks after transplantation through peripheral expansion, whereas the restoration of naive CD4^+^ T cells requires several months to 1–2 years, with full recovery of their number and function being critically dependent on thymic activity [45,46]. Younger patients, with robust thymic function, exhibit faster T-cell recovery and greater repertoire diversity compared with older patients. Consequently, paediatric and young adult transplant recipients display lower risks of infection, relapse and GVHD, which is closely associated with more rapid immune reconstitution [47,48]. In contrast, elderly patients with pronounced thymic involution primarily regenerate memory-phenotype T cells, with restricted TCR diversity. This constrained repertoire may impair immunosurveillance against leukaemia and novel antigens, thereby increasing the risk of post-transplant relapse [49,50].

#### 2.2.2. Factors influencing T-cell immune reconstitution after allo-HSCT

**Figure 1.**
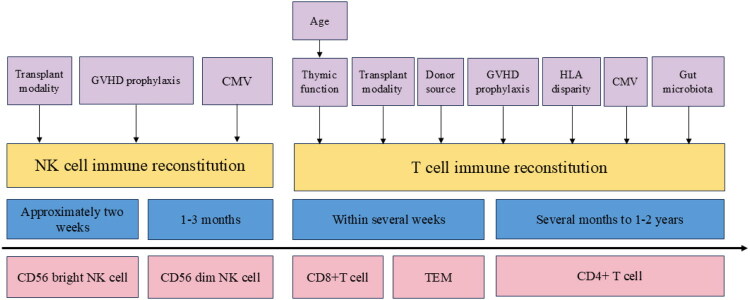
Timeline of immune reconstitution following transplantation. This figure illustrates the sequential pattern and key determinants of T-cell and NK-cell immune reconstitution after transplantation. NK cells recover earliest, followed by T cells. Within the NK-cell compartment, the immature CD56^bright^ subset shows marked recovery within approximately two weeks after transplantation, whereas the mature CD56^dim^ subset gradually reconstitutes over the subsequent 1–3 months. Factors influencing NK-cell reconstitution include the type of transplantation, CMV reactivation and GVHD prophylaxis strategies. For T-cell reconstitution, CD8^+^ T cells and effector memory T cells (TEM) partially recover within several weeks, whereas CD4^+^ T-cell reconstitution is substantially slower, often requiring several months to 1–2 years. Factors affecting T-cell recovery include patient age (particularly thymic function), transplantation modality, donor characteristics, gut microbiota composition and CMV status.

Multiple factors influence post-transplant T-cell immune reconstitution, including patient characteristics and transplantation-related variables [51]. Firstly, patient age and thymic function are intrinsic determinants of T-cell regenerative capacity, with older age corresponding to diminished thymopoiesis. Secondly, transplant modality and donor source significantly impact post-transplant T-cell counts and subset composition: patients undergoing peripheral blood stem cell transplantation, owing to the higher T-lymphocyte content in grafts, demonstrate faster early T-cell recovery compared with bone marrow transplantation, albeit with a higher risk of GVHD [47]. In contrast, cord blood transplantation involves minimal starting T-cell numbers and relies entirely on de novo thymic output, resulting in the slowest recovery [52]. Conditioning regimens and GVHD prophylaxis also play important roles. *In vivo* T-cell depletion strategies, such as antithymocyte globulin (ATG) during conditioning or high-dose post-transplant cyclophosphamide (PTCy), reduce donor T-cell numbers in the early post-transplant phase, thereby delaying T-cell reconstitution [51,53]. A prospective study comparing ATG and PTCy for GVHD prophylaxis revealed that patients receiving ATG exhibited higher lymphocyte counts (including T, B and NK cells) by day 30, whereas the PTCy group subsequently caught up, displaying greater T-cell subset diversity by day 180 [51], highlighting the differential temporal effects of GVHD prophylaxis regimens on immune reconstitution. Furthermore, immune reconstitution is influenced by HLA disparity: mismatched HLA enhances donor T-cell activation, strengthening both GVL and GVHD responses. However, excessive T-cell depletion to mitigate alloreactivity may slow immune reconstitution and increase relapse risk [51]. Microenvironmental and viral factors are also critical. For example, cytomegalovirus (CMV) reactivation drives clonal expansion of virus-specific T cells, dominating the immune space and reshaping the overall T-cell repertoire [54,55]. The gut microbiota further regulates post-transplant immune homeostasis: through its metabolite short-chain fatty acids (SCFAs) promote regulatory T-cell (Treg) IL-10 production and enhance innate lymphoid cell (ILC) and CD4^+^ T-cell IL-22 secretion, reinforcing epithelial barrier integrity and immune equilibrium. Elevated IL-22 further induces antimicrobial peptide expression, modulating microbial community composition and suppressing pathogenic overgrowth, underscoring the microbiota’s key role in transplant-related immune balance via cytokine-mediated pathways [5] (Figure 1).

In patients relapsing after AML transplantation, reduced T-cell numbers or impaired function are frequently observed early post-transplant, implicating inadequate T-cell reconstitution as an important driver of relapse [42]. A multicentre study [42] demonstrated that failure to reach a CD4^+^ T-cell threshold by day 100 was associated with significantly increased relapse risk, whereas early dual reconstitution of CD4^+^ T cells and B cells correlated with reduced relapse. Thus, promoting rapid quantitative and functional recovery of T-cell subsets is critical to minimizing AML relapse and improving survival. In a study by Mathioudaki et al. [24], scRNA-seq of bone marrow T lymphocytes and CD34^+^ cells from post-transplant AML patients identified relapse-associated T-cell signatures. Relapsed patients displayed pronounced inflammatory and immunosuppressive features, including activation of the TNF-α/NF-κB signalling pathway and elevated expression of immunosuppressive transcription factors, consistent with T-cell exhaustion. Integration of single-cell data and functional assays revealed aberrant GPR56 expression in relapsed patients, serving as a dynamic marker of T-cell antigen recognition and cytotoxic engagement. These findings validated the link between T-cell exhaustion and disease relapse.

### 2.3. Immunotherapeutic strategies

#### 2.3.1. Enhancing the GVL effect

Given the pivotal role of donor T cells in mediating the GVL effect, a range of immunotherapeutic approaches targeting T cells have been explored to enhance antileukaemic activity after AML transplantation. Donor lymphocyte infusion (DLI) remains the most classical adoptive T-cell therapy. Introduced in the 1990s, DLI was initially used to induce salvage GVL responses in relapsed post-transplant leukaemia [56]. In chronic myeloid leukaemia (CML), DLI achieves remission in approximately 80% of relapsed cases, but efficacy in AML is limited, with durable complete remission induced in only ∼20–30% of relapsed patients [56,57]. The modest outcomes in AML are attributed to high tumour burden at relapse, rapid proliferation and low immunogenicity of AML cells. Consequently, DLI is increasingly applied in a prophylactic or preemptive context. For high-risk AML patients, low-dose prophylactic DLI administered within the first 3 months post-transplant can target MRD to enhance leukaemia-specific immunity [58]. Alternatively, preemptive DLI at the onset of donor chimerism loss or signs of MRD recurrence yields higher remission rates compared with DLI at overt leukaemia morphology relapse [58,59]. Nevertheless, DLI outcomes remain unsatisfactory, with 2-year survival for AML patients receiving DLI at morphological relapse reported at only ∼25% [57]. To improve efficacy and reduce GVHD risk, multiple strategies are under investigation: (i) *tumour debulking prior to DLI*, using low-intensity chemotherapy or epigenetic agents (e.g. hypomethylating drugs) to control leukaemic proliferation and enhance DLI efficacy; (ii) *cell selection and genetic engineering*, such as selectively enriching donor T cells with antileukaemic potential while depleting GVHD-inducing subsets, or genetically modifying donor T cells (e.g. CAR-T cells) to target AML antigens. Early clinical trials using donor-derived CAR-T cells targeting leukaemia-associated antigens such as CD123 and CLL-1 in relapsed post-transplant AML have demonstrated reduced leukaemic burden and transient remissions in some patients [60,61]. In addition, adoptive T-cell therapies targeting defined leukaemia antigens—such as WT1-specific T cells and TCR-engineered T cells—have shown encouraging activity in early clinical studies of post-transplant AML relapse. In some patients, these approaches have achieved durable molecular remission, supporting the feasibility of antigen-specific precision immunotherapy in this setting [62]. Collectively, T-cell-based therapies are being continually optimized and gradually incorporated into relapse prevention and treatment after AML transplantation.

#### 2.3.2. Attenuation of GVHD

In addition to enhancing T-cell-mediated GVL effects, strategies for controlling GVHD are also crucial for reducing post-transplant relapse in patients with AML. Studies have suggested that mesenchymal stem cells (MSCs) can alleviate GVHD by modulating T-cell function. MSCs exert immunosuppressive effects not only by inhibiting T-cell proliferation, regulating the Th1/Th2 balance, preventing B-cell activation, reducing NK-cell cytotoxicity and influencing the differentiation and maturation of dendritic cells, but also by promoting Treg differentiation through MSC-mediated mitochondrial transfer, thereby directly shaping immune responses and potentially preventing GVHD [63]. Clinical studies have shown that early and repeated infusions of MSCs can reduce the incidence and severity of chronic GVHD [64]. Improving therapeutic outcomes may also be achieved by exploring MSC-based approaches for GVHD prevention. Current perspectives suggest that MSC therapy exerts its effects through immunomodulatory properties, anti-inflammatory activity and paracrine signalling, although these functions are influenced by factors such as the microenvironment and MSC immune phenotype [65,66]. Accordingly, ongoing efforts aim to optimize MSC-based therapies to address limitations in clinical application. These strategies include identifying MSC subsets with enhanced immunosuppressive capacity, engineering or modifying MSCs, reprogramming their glycolytic metabolism and combining MSCs with pharmacologic agents to improve therapeutic efficacy [66]. Among these approaches, MSC-derived extracellular vesicles have emerged as a major research focus due to their immunomodulatory activity, ease of isolation, strong expandability *in vitro*, small size and ability to traverse most physiological barriers [67]. However, issues related to dose dependency, potential adverse effects and reliable methods for monitoring therapeutic efficacy still require further investigation [68].

Circadian rhythms in cytokine levels also influence the development of GVHD. Zhu and coauthors demonstrated through animal models and retrospective clinical analysis that administering stem cell infusions during the active phase of the recipient’s circadian cycle significantly reduces the incidence and severity of aGVHD and improves survival. Mechanistic analyses revealed that circadian oscillations modulate the rhythmic fluctuation of inflammatory cytokines following myeloablative conditioning—particularly the diurnal variation in IL-1α—which in turn regulates the activation, proliferation and cytokine production of donor-derived T cells. The study further showed that blocking IL-1α signalling partially alleviates aGVHD, suggesting that IL-1α serves as a key mediator linking circadian regulation to alloimmune responses. These findings indicate that optimizing the timing of stem cell infusion based on circadian biology may hold potential clinical value for the prevention and management of aGVHD [69].

#### 2.3.3. Immune checkpoint inhibitors

Immune checkpoint inhibitors (ICIs) have also gained attention in post-transplant AML. Residual AML cells and the post-transplant microenvironment frequently exploit inhibitory molecules such as PD-L1 and B7-H3 to drive donor T-cell exhaustion and evade immune clearance [70]. To counteract this, ICIs including anti-CTLA-4 and anti-PD-1/PD-L1 antibodies have been tested. In a phase I trial, the CTLA-4 inhibitor ipilimumab achieved remission in ∼30% of refractory relapsed AML patients post-allo-HSCT, including regression of extramedullary disease, demonstrating restored T-cell antitumour activity [71]. However, such therapies may disrupt transplant tolerance, thereby increasing the risk of severe GVHD and other immune-related toxicities. Trials combining PD-1 blockade with hypomethylating agents in relapsed/refractory AML after allo-HSCT reported MRD negativity or disease control in subsets of patients, but also documented significant GVHD incidence and transplant-related mortality [72]. Thus, ICI use post-transplant requires careful risk–benefit consideration, and current applications are largely restricted to relapsed/refractory cases, with prophylactic use remaining inconclusive.

Emerging immunomodulatory strategies are also under exploration. Low-dose IL-2 therapy has been proposed to selectively expand donor-derived Tregs to mitigate GVHD, while early withdrawal of immunosuppressants aims to ‘release’ donor T-cell activity while preserving GVL [73]. Although these approaches hold potential for further optimizing T-cell-mediated GVL effects, robust clinical evidence is still required.

## 3. NK cells in allogeneic haematopoietic stem cell transplantation for AML

### 3.1. Cytotoxic mechanisms

NK cells, a lymphocyte subset of the innate immune system, play a distinctive role in transplant immunology for AML. Unlike T cells, NK-cell cytotoxicity does not dependent on antigen-specific receptor recognition but is instead governed by the principles of ‘missing self’ and stress-induced signals [74]. NK cells express both inhibitory and activating receptors: inhibitory receptors (e.g. the KIR family, NKG2A) recognize self-MHC class I molecules and deliver inhibitory signals to prevent attacks on healthy autologous cells. When target cells lose or downregulate MHC class I (‘missing self’), inhibitory signalling diminishes, rendering NK cells more prone to activation [74–76]. AML cells frequently exhibit low HLA-I expression to evade T-cell immunity, a ‘camouflage’ strategy that paradoxically makes them prime targets for NK cells [70]. In addition, stressed leukaemic cells upregulate NKG2D ligands such as MICA/B and ULBP family proteins, which engage NK-cell receptors like NKG2D, thereby triggering NK cytotoxicity [74,76,77]. Once activated, NK cells induce target-cell lysis via perforin/granzyme release and initiate apoptosis through FasL and TRAIL pathways. Furthermore, NK cells secrete cytokines such as IFN-γ and TNF-α, which contribute to antileukaemic immunity and modulate subsequent adaptive responses [78]. However, NK cells do not maintain sustained cytotoxic activity. Studies [79] have shown that GARP expressed on CD4^+^ T cells promotes the activation of TGF-β1, and the activated TGF-β1 markedly suppresses mTORC1 activity and mitochondrial oxidative phosphorylation in NK cells. This suppression impairs NK-cell proliferation and cytotoxicity, thereby compromising NK-cell-mediated immune surveillance against leukaemic cells, diminishing their antileukaemia activity and ultimately contributing to early AML relapse following allo-HSCT.

#### 3.1.1. NK-cell KIR mismatch mechanisms

In the allo-HSCT setting, donor NK recognition of recipient AML cells is particularly important. When donor NK inhibitory KIRs fail to find cognate HLA ligands on recipient cells (i.e. KIR-ligand mismatch), NK cells are released from inhibition and acquire potent cytotoxicity against recipient leukaemic cells [76,80]. Ruggeri et al. [80] demonstrated that in haploidentical transplants, KIR-ligand mismatching significantly reduced relapse rates and improved survival compared with matched settings. Another study [81] revealed that KIR and HLA genotypes associated with low-affinity interactions correlated with reduced relapse rates in AML patients undergoing autologous transplantation. In the study by Boudreau et al. [82], analysis of the matching status between a common donor KIR receptor (KIR3DL1) and recipient HLA-B in allogeneic haematopoietic stem cell transplantation revealed that strong KIR3DL1–HLA-B inhibitory interactions result in excessive NK-cell inhibition, which is associated with an increased risk of relapse and reduced survival. Clinically, donor selection strategies incorporating KIR mismatching with recipient HLA may confer enhanced NK-mediated GVL activity [82].

Beyond leukaemia killing, donor NK cells may influence GVHD. Studies suggest that donor NK alloreactivity can eliminate recipient antigen-presenting cells, thereby reducing opportunities for donor T cells to attack healthy tissues and potentially mitigating GVHD [83]. Preclinical and clinical evidence indicates that NK cells can reduce or prevent GVHD without compromising GVL activity [80,83,84], suggesting that NK cells may help dissociate these two immunological phenomena. Overall, NK cells exert innate antileukaemic activity via ‘missing self’ recognition and activating receptor signalling. In allo-HSCT, donor NK cytotoxicity can be magnified by HLA disparities, contributing to effective immune surveillance. However, the clinical utility of KIR mismatch remains debated. An external validation study [85] reported no significant association between different KIR3DL1–HLA-B matching combinations and relapse or survival. The investigators concluded that neither KIR mismatch nor KIR genotype is significantly correlated with post-transplant relapse or survival and therefore should not be used as a criterion for donor selection, challenging the conclusions of earlier studies. One hypothesis proposed to explain the failure to replicate prior findings is that differences in patient characteristics and transplant procedures across cohorts may influence outcomes. Importantly, the study highlighted that AML relapse more commonly occurs through immune evasion mechanisms such as HLA class II downregulation, whereas loss or downregulation of HLA class I is rare. Such patterns may fail to trigger NK-cell activation. In addition, the strong peptide dependency and inter-individual variability in KIR–HLA interactions further complicate the prediction of KIR-mediated immune responses. Although this study did not identify a consistent KIR-based relapse prediction model across multiple subgroups, the potential value of KIR mismatch cannot be fully excluded, particularly within specific windows of immune reconstitution, specific transplant settings or distinct leukaemic immunophenotypes. Therefore, whether selecting donors with favourable KIR profiles can help predict post-transplant outcomes remains an open question that warrants further investigation. Schetelig et al. [85], emphasized that future research should validate different KIR and KIR-ligand or haplotype hypotheses and incorporate cross-cohort collaborations to determine whether KIR-based donor selection can improve allo-HSCT outcomes. They further suggested studying the differential roles of infused mature NK cells versus host-matured NK cells in NK-cell-mediated graft-versus-host reactions to deepen mechanistic understanding.

### 3.2. Immune reconstitution

NK cells are among the earliest lymphocyte subsets to reconstitute following transplantation. Unlike T and B cells, which require months to recover, donor-derived NK cells often reach substantial numbers within weeks, contributing to early protection against infection and leukaemia [51,55,80,86]. By day 14 post-transplant, high levels of donor NK chimerism correlate with significantly reduced relapse rates [80]. Under non-myeloablative conditions, patients with early complete donor NK chimerism showed lower relapse risks compared with those with incomplete chimerism [82]. Subset dynamics are also important: early post-transplant NK compartments are dominated by immature CD56^bright NK cells, which are gradually replaced by mature CD56^dim NK cells with restored cytotoxic function [55,87]. Functionally, CD56^bright NK cells exhibit strong proliferative and cytokine-secreting capacity, whereas CD56^dim NK cells display higher cytolytic activity with reduced proliferative potential [88].

#### 3.2.1. Factors Influencing NK-cell immune reconstitution after allo-HSCT

NK-cell reconstitution is influenced by transplant modality and GVHD prophylaxis. ATG-based conditioning, by depleting T cells, reduces competition for cytokines such as IL-7 and IL-15, enabling more robust NK recovery while delaying CD4^+^ and CD8^+^ T-cell reconstitution [51]. Conversely, PTCy suppresses the recovery of both T and NK cells [51]. Charrier et al. [89] reported that NK counts reached normal levels by 1 month after both cord blood and bone marrow transplantation, but cord blood recipients exhibited significantly higher NK counts at 3, 9 and 12 months, underscoring the influence of graft source. Viral reactivation also shapes NK immunity. Schäfer et al. [55] found that CMV reactivation promoted expansion of mature, highly cytotoxic NK subsets, reflecting a strongly activated immune milieu (Figure 1).

Early NK recovery provides crucial leukaemic surveillance during the interval before T-cell reconstitution, forming a frontline defense in the immediate post-transplant period. Clinical evidence supports this: patients with robust NK reconstitution by day 32 exhibited improved 3-year progression-free survival, lower relapse and reduced mortality [90]. Thus, strategies to promote early NK recovery represent a key avenue for improving allo-HSCT efficacy in AML (Figure 1).

### 3.3. Immunotherapeutic strategies

#### 3.3.1. Enhancing the GVL effect

Because NK cells possess the unique ability to kill tumour cells independently of antigen presentation and are associated with relatively low risks of adverse reactions such as GVHD, a variety of NK-cell-based adoptive immunotherapies have been explored in recent years for the treatment and prevention of AML after transplantation. Donor NK-cell infusion represents one important therapeutic approach. NK cells can be isolated from donor peripheral blood, activated and expanded *ex vivo* and subsequently infused into recipients to enhance early post-transplant antileukaemic activity [91]. In a phase I clinical trial, Ciurea et al. [91] infused IL-21-costimulated, *ex vivo*-expanded donor NK cells into high-risk AML patients following haploidentical transplantation. The study demonstrated feasibility and safety: even high-dose NK infusions did not induce significant adverse reactions or GVHD, and patients showed a trend toward reduced 2-year relapse rates compared with historical controls. Although this trial primarily established safety, it provided proof-of-concept for donor NK adoptive therapy, with efficacy requiring confirmation in subsequent controlled studies.

In relapsed patients, NK adoptive transfer has also shown promise. A comparative study analysing cytokine-induced killer (CIK) cells (a mixed T- and NK-cell population) versus NK cells for relapsed leukaemia found overall remission rates of 82.8% in the NK group, significantly higher than 48.9% in the CIK group, with lower rates of newly developed GVHD (∼11%) in NK recipients [42]. These findings suggest that pure NK-cell therapy may offer stronger antileukaemic activity with favourable safety. Dolstra et al. [92] reported an early-phase study in 10 elderly AML patients in morphological remission who received haematopoietic stem and progenitor cell-derived NK (HSPC-NK) cell infusions. The treatment was well tolerated, with no GVHD or significant toxicity, highlighting HSPC-NK adoptive transfer as a potentially ‘off-the-shelf’ cellular immunotherapy for AML.

#### 3.3.2. CIML-NK and CAR-NK

Despite encouraging results, current NK therapies face challenges such as limited persistence and difficulty achieving *in vivo* expansion. To overcome these barriers, innovative strategies are under investigation. One such approach is the use of cytokine-induced memory-like NK (CIML-NK) cells. Inflammatory cytokines can confer memory-like properties to both murine and human NK cells even in the absence of antigen exposure. These CIML-NK cells exhibit enhanced cytotoxicity, increased cytokine production and prolonged persistence upon re-encounter with antigens or related stimuli [93]. Short-term preactivation with IL-12, IL-15 and IL-18 endows NK cells with a ‘memory-like’ phenotype, reprogramming their transcriptional, epigenetic and metabolic state through activation of the JAK-STAT and PI3K–AKT–mTOR signalling pathways [94]. Mechanistically, IL-15 bound to IL-15Rα on antigen-presenting cells is trans-presented to the IL-2/IL-15Rβγ heterodimer on NK cells, inducing JAK-STAT signalling, upregulating anti-apoptotic proteins BCL-2 and MCL-1 as well as c-Myc and activating the adaptor protein Shc, which further engages PI3K–AKT–mTOR to enhance NK metabolic activity and function [94]. Clinically, Fehniger and coauthors [95] tested CIML-NK cells in relapsed/refractory AML patients, observing substantial leukaemic clearance in ∼50% of cases, with some achieving complete remission. These cells persisted for months *in vivo* and maintained functionality, establishing durable immune pressure. CIML-NK therapy is now being evaluated in phase II trials.

Another promising direction is CAR-NK-cell therapy. Similar to CAR-T approaches, NK cells are engineered with chimeric antigen receptors (CARs) to recognize AML-associated targets such as CD33 and CD123. CAR-NK cells have the advantage of not inducing GVHD and may be derived from allogeneic donors or even established cell lines [96]. Several early-phase clinical trials [60,97–99] have shown that CAR-NK therapy exerts potent antileukaemic effects in AML, further confirming both feasibility and safety.

#### 3.3.3. ICIs

NK checkpoint inhibition and functional enhancement are also emerging research focuses. NK cells in AML often exhibit exhaustion phenotypes, characterized by high expression of inhibitory receptors such as TIGIT and NKG2A [100]. Therapeutic antibodies targeting these molecules, including monalizumab (anti-NKG2A) [101] and anti-TIGIT antibodies [102], are being developed to release NK cells from suppression. Preclinical studies demonstrated that TIGIT knockout enhanced NK cytotoxicity against tumour cells, and combining TIGIT knockout with Fc-active anti-TIGIT antibodies further improved cytotoxicity, metabolic adaptability and resistance to fratricide among expanded NK cells [102]. André et al. [101] showed that blocking NKG2A boosted both NK and CD8^+^ T-cell effector functions in mice and humans, enhancing antitumour immunity. Monalizumab thus represents a novel checkpoint inhibitor mechanism that augments both NK- and T-cell activity. Several early-phase clinical trials are currently evaluating these NK-directed checkpoint inhibitors in AML. Other studies [79] have demonstrated that inhibition of the TGF-β1 signalling pathway using the TGF-β1 inhibitor galunisertib can enhance the effector functions of bone marrow-derived NK cells from patients with early relapsed AML, thereby restoring NK-cell-mediated antileukaemic responses in xenograft mouse models of leukaemia.

Finally, bispecific and multispecific therapeutics are being investigated. Examples include bispecific antibodies that simultaneously engage NK cells and leukaemia cells (e.g. anti-CD16 × CD33 bispecific antibodies) and trispecific killer engagers incorporating IL-15 moieties, which further stimulate NK survival and cytotoxicity. Preclinical studies have shown that such agents promote NK-mediated killing of AML cells and may represent next-generation strategies [103].

In summary, NK-cell immunotherapy is evolving from conventional adoptive transfer toward engineered cellular products and combination approaches. These novel strategies hold promise for strengthening post-transplant immune surveillance in AML, thereby reducing relapse and improving long-term outcomes.

## 4. Interactions and synergistic effects between T cells and NK cells

In post-transplant antileukaemic immunity, T cells and NK cells act as two principal effector populations that not only perform distinct functions but also influence one another, resulting in synergistic effects. First, they display temporal complementarity: NK cells, which reconstitute rapidly after transplantation, serve as a ‘first-response force’ during the immunologic nadir, eliminating residual leukaemia and suppressing its expansion, while T cells gradually reconstitute later to provide long-term immunosurveillance [51]. This relay between innate and adaptive immunity contributes to continuous suppression of leukaemic clones throughout the post-transplant course.

Functionally, positive cross-regulation also occurs between the two populations. Donor T cells secrete cytokines such as IL-2 and IL-15 that not only sustain their own proliferation but also promote NK-cell survival and activation [70,94]. IL-15, in particular, is critical for the maintenance of NK cells and memory CD8^+^ T cells. One study demonstrated that patients with lower plasma IL-15 levels early after transplantation were more prone to leukaemia relapse [104], underscoring the importance of a sufficient cytokine milieu for effective T-cell and NK-cell antileukaemic synergy. Conversely, NK cells regulate T cells responses primarily by constraining harmful alloreactivity. As noted previously, NK cells can eliminate residual recipient antigen-presenting cells, thereby reducing opportunities for donor T cells to be activated against healthy tissues and trigger GVHD [32,83]. This ‘scavenger’ function of NK cells is particularly valuable in highly HLA-mismatched transplants, creating a safer immunologic environment in which reconstituting T cells can focus their activity on leukaemic cells rather than normal host tissues.

Clinical studies further support the importance of T-cell and NK-cell synergy. During DLI, the co-infusion of donor NK cells (i.e. ‘composite lymphocyte infusion’) or sequential administration of NK and T cells, has been shown to produce synergistic cytotoxicity with potential for improved efficacy without increased GVHD risk [105,106]. In a phase II trial [107], haploidentical transplantation using memory T-cell-rich grafts combined with NK therapy in paediatric leukaemia resulted in rapid immune reconstitution, maximized GVL effects and GVHD episodes that were manageable and rapidly resolved. The study reported excellent survival outcomes, with 3-year overall survival and event-free survival rates of 92% and 88%, respectively, in patients transplanted in first complete remission (CR1). These findings highlight the cooperative role of NK and T cells in immune reconstitution, enhancement of the GVL effect and mitigation of GVHD.

**Figure 2.**
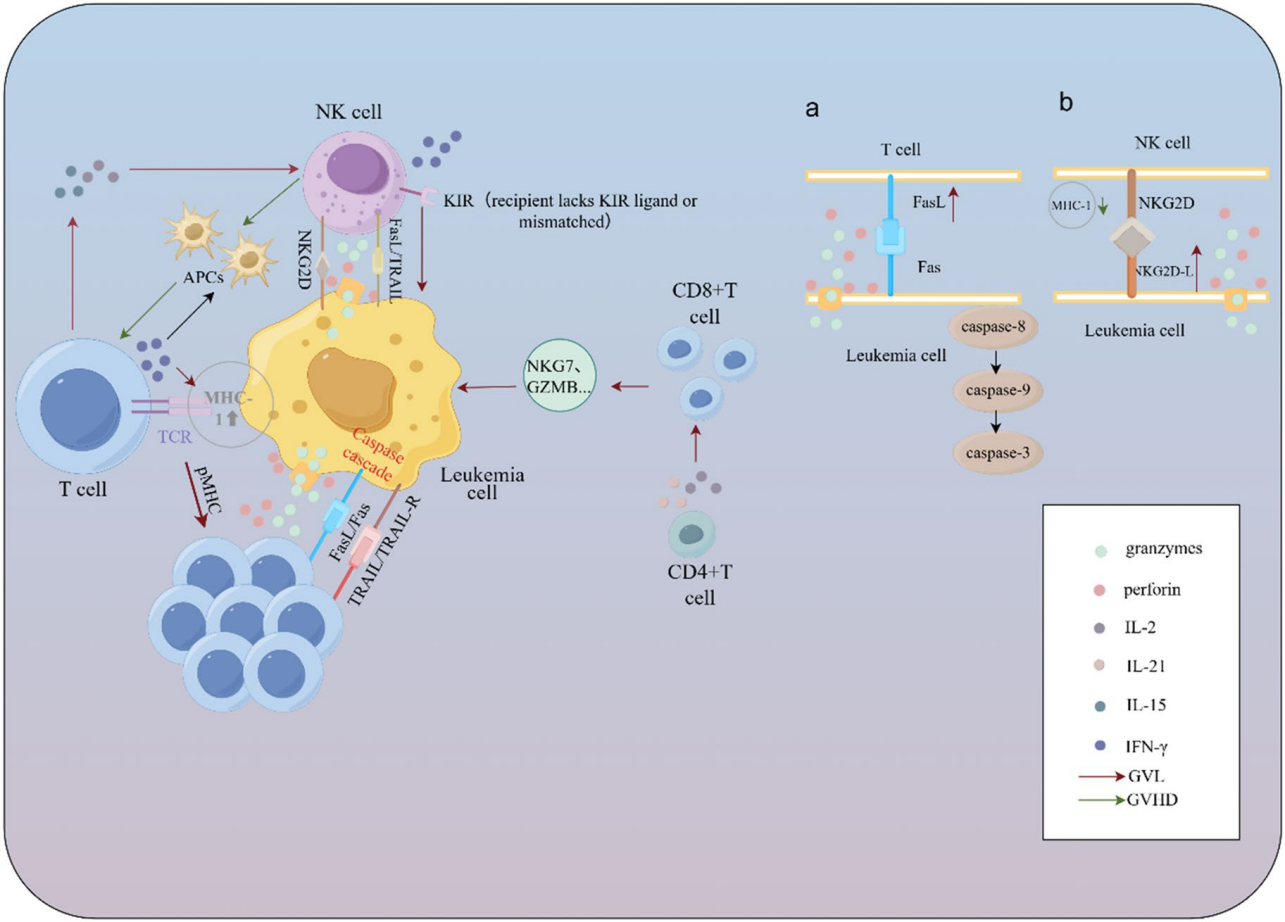
Antileukemic effects of T cells and NK cells. (a.Fas-mediated apoptosis signaling pathway by T cells; b. NKG2D signaling pathways of NK cells.). This figure illustrates the coordinated antileukaemic mechanisms of donor T cells and NK cells after allo-HSCT. Donor T cells recognize leukaemia-associated antigens and mediate cytotoxicity through perforin–granzyme, FasL/Fas and TRAIL pathways, while IFN-γ secretion enhances MHC class I expression and antigen presentation. CD4^+^ T-cell-derived cytokines (e.g. IL-2, IL-21) support the activation and expansion of cytotoxic CD8^+^ T cells, strengthening the GVL response. AML cells that downregulate MHC class I become susceptible to NK-cell killing via NKG2D, perforin–granzyme and FasL/TRAIL pathways; KIR mismatch further augments NK-cell activity. Donor T-cell-derived IL-2 and IL-15 promote NK-cell survival and activation, whereas NK-cell clearance of residual host APCs helps limit excessive donor T-cell activation and reduces GVHD risk.

In summary, T cells and NK cells do not operate in isolation in AML transplant immunity. NK cells compensate for the lag in T-cell reconstitution while limiting alloreactive responses, thereby enabling T cells to more effectively exert their GVL function. Meanwhile, T-cell-derived cytokines and long-lasting memory responses support NK-cell persistence and functionality. Elucidating the mechanisms underlying T-cell and NK-cell interactions and translating these insights into optimized cellular therapy strategies hold promise for further enhancing the durability and selectivity of the GVL effect (Figure 2).

## 5. Challenges and future perspectives

**Figure 3.**
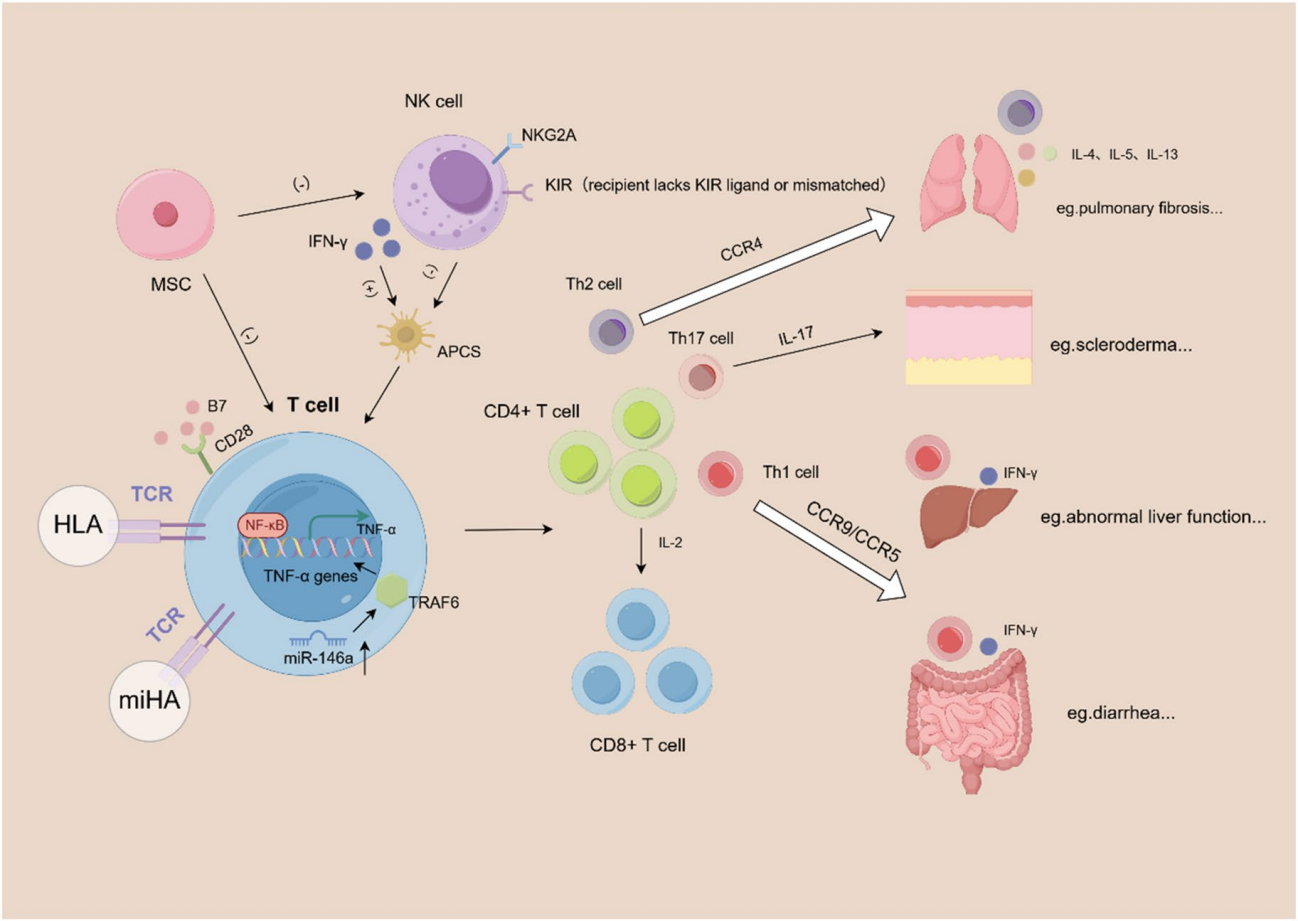
Immunological crosstalk of T cells and NK Cells in GVHD pathogenesis. Following allo-HSCT, T cells recognize either MHC molecules or miHAs and subsequently become activated and differentiate into CD4^+^ and CD8^+^ effector subsets and mediate tissue injury through FasL/Fas signalling or perforin–granzyme cytotoxic pathways, with B7 and CD28 costimulation further enhancing their activation. miR-146a, by targeting TRAF6 and modulating NF-κB dependent transcription of TNF-α, can attenuate GVHD. CD4^+^ T cells further differentiate into Th1, Th2 and Th17 subsets, which migrate to the gastrointestinal tract and liver, the lungs or the skin, respectively, and contribute to GVHD-associated tissue damage through the secretion of their characteristic cytokines. NK cells exhibit a dual role in GVHD: their inhibitory receptors (such as KIR and NKG2A) prevent them from attacking normal host tissues and by eliminating recipient antigen-presenting cells they reduce excessive donor T-cell activation; however, their production of IFN-γ can enhance antigen presentation and indirectly exacerbate GVHD. In addition, MSCs can suppress T-cell proliferation and reduce NK-cell cytotoxicity, thereby exerting broad immunoregulatory effects that contribute to the prevention of GVHD.

Despite significant advances in the immunological study of AML allogeneic transplantation in recent years, several critical challenges remain unresolved. Firstly, the fundamental distinction between GVL and GVHD has not been fully elucidated. It remains unclear which antigens and cellular pathways are ‘beneficial’ in mediating GVL and which are ‘harmful’ in driving GVHD. Most minor antigens recognized by donor T cells are expressed not only on leukaemic cells but also on normal tissues, thus capable of mediating both GVL and GVHD (Figure 3).

Future research should not only focus on identifying antigens that are specifically or highly expressed in AML but minimally expressed in normal tissues, but also leverage spatial transcriptomics to dissect the immunological microenvironmental differences between GVL and GVHD. These approaches may help identify key cellular populations that promote GVL without inducing GVHD, map the spatial organization and interactions of immune cells within GVL and GVHD target tissues [108]. Such targets could guide the development of selective immunotherapies—such as antigen-specific T cells or monoclonal antibodies—designed to enhance GVL while minimizing GVHD. Current strategies including TCR-engineered T cells and antigen-specific vaccines against leukaemia-associated targets are in early stages, with the ultimate goal of discovering true ‘GVL antigens’ to achieve precise immune targeting.

Secondly, immune evasion by leukaemia poses major obstacles to effective GVL. Studies show that nearly two-thirds of relapsed AML cases after transplantation exhibit clear immune escape mechanisms, such as loss of HLA alleles required for donor recognition (particularly selective HLA loss mutations in haploidentical transplants) or overexpression of inhibitory molecules such as PD-L1 and B7-H3, which drive functional exhaustion of donor T/NK cells [70]. In the study by Kong et al. [109], PD-1^hiTIM-3^+^ cells were found to be strongly associated with relapse after allo-HSCT. These subsets were enriched in both CD4^+^ and CD8^+^ T-cell compartments of relapsed patients, exhibiting markedly reduced secretion of IL-2, TNF-α and IFN-γ—hallmarks of T-cell exhaustion. Importantly, expansion of these subsets preceded clinical relapse, highlighting their potential as early predictive biomarkers of post-transplant recurrence. AML relapse is also commonly associated with downregulation of HLA class II, upregulation of T-cell inhibitory ligands such as PD-L1 and alterations in the tumour microenvironment [109–111], underscoring the multifaceted nature of immune escape. Current countermeasures include: (i) for relapse due to HLA loss, considering re-transplantation with a different donor whose T-cell repertoire can recognize the remaining HLA alleles [83]; (ii) for leukaemias with high expression of checkpoint molecules, timely introduction of checkpoint inhibitors or related therapies to release the ‘brakes’ on effector cells [71,72,112] and (iii) for immunosuppressive microenvironments characterized by factors such as IDO or arginase secretion, exploring the use of targeted inhibitors or gene-editing approaches to eliminate these escape mechanisms and restore GVL activity. To systematically identify key genes involved in immune evasion in AML, CRISPR-based genome-wide screening can be used to uncover core regulatory factors that reduce immune sensitivity in leukaemic cells. Such screening typically begins with the establishment of a stable Cas9-expressing AML cell line, followed by transduction of these Cas9^+^ cells with a pooled CRISPR sgRNA library at a low multiplicity of infection, ensuring that each cell carries only a single sgRNA. The resulting cell population is then subjected to defined selection conditions, during which sgRNAs targeting essential genes become depleted, whereas those conferring a survival advantage are enriched. By comparing sgRNA abundance between the post-selection population and the baseline library through deep sequencing, genes that play critical roles in the biological process of interest can be identified [113]. These insights can subsequently guide the design of targeted strategies to counteract immune-evasion mechanisms in AML.

Thirdly, novel immunotherapies must strike a delicate balance between efficacy and safety. Potent strategies such as CAR-T and CAR-NK therapies face challenges in AML due to difficulties in antigen selection and risks of on-target off-tumour toxicity. Identifying ideal target antigens and implementing ‘safety switches’ remain priorities. Current clinical trials are evaluating CAR-T/NK therapies against AML-associated antigens such as CD33 and CLL-1, but since CD33 is also expressed on normal myeloid cells, collateral damage to normal haematopoiesis remains a significant hurdle [60]. One potential solution is to apply CAR-T therapy in relapsed post-transplant patients, where normal haematopoiesis has already been donor-derived, thereby reducing the impact on host haematopoietic reserves. Additionally, suicide genes can be engineered into CAR-T cells to allow rapid elimination of effector cells in the event of severe adverse effects. For adoptive T-cell therapies, strategies to mitigate GVHD risk are equally important. Gene-editing technologies enabling removal of donor T-cell αβ TCRs or incorporation of suicide switches have been developed to preserve GVL activity while permitting pharmacologic ablation if GVHD arises [114]. Such approaches hold promise for improving the safety profile of cellular immunotherapy (Table 1).

**Table 1.** Common antigen targets for CAR-T/NK cells in AML.

Target Ag	Expression on AML	Expression on healthy cells	Clinical trial drugs	Potential toxicity risk	Solutions	References
CD33	>90%	Multipotent myeloid precursors, unipotent colony-forming cells, maturing granulocytes, monocytes, peripheral granulocytes and resident macrophages, Kupffer cells and hepatocyte	e.g. Ozogamicin,lintuzumab-CD28/CD3Z (H195HLh28Z)	Myelosuppression, hepatotoxicity	1. Membrane-proximal targeting with a high-affinity single-chain variable fragment (scFv) raised through epitope-specific immunization; 2. Inhibition of cytokine release syndrome (CRS) through anti-TNF-α and/or anti-IL-6 therapy; 3. Use the Universal CARs (UniCARs).	[115–117]
CD123	78–89%	Multipotent progenitors, myeloid progenitors, lymphoid progenitors and immature blood cells and endothelial cells	e.g. UCART123	Haematopoietic inhibition	1. DNA methyltransferase inhibitors synergistically with CAR-T cells; 2. Use dual-CAR cells that co-express two independent types of CARs specific for different antigens or a bispecific tandem CAR (TanCAR-T cell); 3. Genetically engineered CAR-T cells anti-CD123 which do not express TCRαβ were constructed; 4. Use the UniCARs; 5. CAR-T-cell depletion following eradication of leukaemic cells; 6. Combine with safety switches.	[117–119]
CLL-1	85–92%	normal bone marrow cells, such as granulocytes, monocytes, macrophages and dendritic cells	e.g. Anti-CLL-1 CAR-T cells, KITE-222,Anti-CLL-1 CAR-T cells with PD-1 KO	CRS, cytotoxic activity against mature myeloid cells, agranulocytosis	1. Combine with a safety switch based on inducible caspase 9 (iC9); 2. Combine with anti-TNFα antibodies and security system; 3. Combine with programmed death receptor 1 (PD-1) silencing.	[117,119]
FLT3	54–92%	HSCs and myeloid cells	e.g. Anti-FLT3 CAR-T cells, AMG 553, TAA05	Depletion of the HSPC	1. Manufacture the cells under conditions that promote a memory stem cell phenotype; 2. Combine with the FLT3 inhibitor; 3. Combine with safety switches; 4. CAR-T-cell depletion following eradication of leukaemic cells and allo-HSCT to reconstitute the haematopoietic system.	[117,120,121]
NKG2DL	The expression level is relatively low	monocyte-like myeloid-derived suppressor cells, gut epithelium; (in healthy non-neoplastic cells including stem cells remains largely absent or undetectable)	e.g. CYAD-01	The possibility of ligand-upregulation on healthy cells, the induction of an inflammatory feedback loop	1. Treatment with the HDAC inhibitor upregulates NKG2DL in AML; 2. Design CARs that target two cell membrane molecules;	[119,122–124]

Lastly, personalized immune monitoring and intervention will represent an important future direction. Using high-throughput sequencing and related technologies to track immune cell dynamics in post-transplant patients can enable timely assessment of T-cell clonal diversity, functional status and NK receptor expression profiles, thereby predicting relapse risk and guiding clinical interventions. Another approach involves the development of biomarkers for early prediction and timely intervention. For example, scRNA-seq-based analyses distinguishing T-cell profiles between AML patients in remission and those who relapsed have identified GPR56 expression as a potential indicator of donor T-cell capacity to recognize and eliminate residual leukaemic stem cells. However, further studies are needed to validate the value of GPR56 as an early biomarker of therapeutic response [24]. Circulating tumour DNA (ctDNA) has also been proposed as a valuable non-invasive prognostic biomarker for patients with AML or MDS undergoing allo-HSCT [125]. In addition, the combined ST2 and TNFR1 biomarker model has been shown to effectively predict the risk of non-relapse mortality within 6 months after allo-HSCT. This model is not restricted by baseline clinical characteristics or specific complications and can be dynamically monitored during the treatment course of aGVHD [126]. Machine learning and artificial intelligence are increasingly becoming powerful tools for improving disease management. By enabling the construction of more accurate prognostic and predictive models, these technologies can substantially enhance the precision and efficiency of diagnosis, risk stratification, therapeutic decision-making and disease monitoring [127]. In the future, as immunomonitoring datasets continue to expand—including single-cell transcriptomics, TCR/BCR repertoire profiling and diverse biomarker signatures—the integration of AI and machine-learning approaches is expected to facilitate comprehensive analyses of these complex data. Such advances may deepen our understanding of immune status and immune-evasion mechanisms in patients with AML and ultimately support the development of more targeted, synergistic and personalized combination immunotherapies [128–130]. For example, a significant decline in WT1-specific T-cell numbers or the emergence of exhausted T-cell phenotypes in peripheral blood may warrant early interventions, such as prophylactic DLI or administration of immunostimulatory agents. Similarly, subtle declines in donor chimerism can be evaluated in conjunction with MRD detection and immunologic profiling to distinguish between leukaemic relapse and immune rejection, enabling tailored management strategies.

In summary, future research must integrate immunology, genomics and other multidisciplinary approaches to unravel mechanistic complexities of AML transplant immunity and to develop more precise and effective immunotherapeutic strategies. These efforts hold the promise of improving cure rates for AML following allo-HSCT, enabling more patients to achieve long-term survival with better quality of life.

## 6. Summary

T cells and NK cells together constitute the cornerstone of antileukaemic immunity after allo-HSCT in AML. Donor T cells mediate GVL effects that eradicate residual leukaemia and represent the principal curative mechanism of transplantation, though their potential to induce GVHD remains a major concern. NK cells, by contrast, provide rapid early antileukaemic activity and hold the unique potential to mitigate GVHD. The immune reconstitution process post-transplant is highly complex and shaped by numerous factors; the quantitative and functional recovery of T and NK cells directly determines relapse risk and patient survival.

Over recent years, immunotherapeutic strategies have expanded from classical DLI to advanced modalities such as CAR-T/CAR-NK therapies and memory-like NK cells, enriching the therapeutic armamentarium. However, critical challenges persist, including balancing GVL with GVHD, overcoming leukaemic immune escape and ensuring treatment safety. Future research should focus on dissecting underlying mechanisms, optimizing therapeutic designs and leveraging the synergistic antileukaemic potential of T and NK cells. Ultimately, the goal is to improve long-term disease-free survival and quality of life for AML patients.

Only through the integration of immune monitoring, single-cell omics and cellular engineering can the cooperative potential of T and NK cells be transformed into predictable, controllable GVL effects—advancing allo-HSCT from a ‘probabilistic cure’ toward a paradigm of ‘precision cure’.

## Data Availability

Data sharing is not applicable to this article as no datasets were generated during the current study.
